# Demographic and clinical characteristics of hypertensive patients in the internal medicine outpatient clinic of a university hospital in Rio de Janeiro

**DOI:** 10.1590/S1516-31802004000300003

**Published:** 2004-05-06

**Authors:** Elizabeth Silaid Muxfeldt, Armando da Rocha Nogueira, Gil Fernando Salles, Kátia Vergetti Bloch

**Keywords:** Hypertension, Cardiovascular diseases, Blood pressure, Ambulatory blood pressure monitoring, High blood pressure, Hipertensão, Controle, Pressão arterial, Monitorização ambulatorial da pressão arterial, Doenças cardiovasculares

## Abstract

**CONTEXT:**

Hypertension is one of the most important cardiovascular risk factors but its control is still a challenge for physicians all around the world. For blood pressure control to be improved, it is important to guarantee the quality of attendance provided for hypertensive patients, especially in teaching hospitals, where future physicians are being trained.

**OBJECTIVE:**

To characterize the profile of hypertensive patients attending the internal medicine out-patient clinic of a university hospital in Rio de Janeiro, describing their cardiovascular risk and identifying flaws in the treatment provided for severely hypertensive patients, in order to implement an arterial hypertension management program.

**TYPE OF STUDY:**

A descriptive cross-sectional population-based study.

**SETTING:**

Hospital Universitário Clementino Fraga Filho, Universidade Federal do Rio de Janeiro.

**METHODS:**

The study was carried out over a period of four months, involving all the hypertensive patients under treatment in the outpatient unit. The attending physician obtained information relating to demographic features, cardiovascular risk factors, target organ damage, blood pressure levels, therapeutic regimens and compliance with treatment. Means and the respective standard deviations and proportions were used to describe the distribution of patient data.

**RESULTS:**

Of the total number of patients seen, 24.2% (1,699 patients) were hypertensive. Women accounted for 65.0% of the patients. The mean age was 63.9 years. Dyslipidemia (49.2%) and diabetes (29.8%) were the most frequently reported risk factors and heart disease was the most prevalent end-organ damage. Seventy percent of the patients were classified as high cardiovascular risk. In spite of the high intensity treatment provided for the most severe patients (19.4% on a regimen of 3 or more antihypertensive drugs), the rate of blood pressure control was low (27%).

**CONCLUSIONS:**

The patients with arterial hypertension under treatment at the university hospital had a profile of high cardiovascular risk and poor blood pressure control. Greater effort for improving hypertension control is needed, since this is the only way to reduce the morbidity and mortality rates of cardiovascular diseases.

## RESUMO

**CONTEXTO:**

A hipertensão é um dos mais importantes fatores de risco cardiovasculares e a dificuldade de controle dos níveis tensionais é um desafio aos médicos em todo o mundo. Para melhorar o controle da pressão arterial é importante garantir a qualidade da assistência aos pacientes hipertensos, principalmente em hospitais de ensino nos quais futuros médicos estão sendo formados.

**OBJETIVOS:**

Caracterizar o perfil dos hipertensos do ambulatório de Clínica Médica de um hospital universitário no Rio de Janeiro e descrever o risco cardiovascular desses pacientes, identificando as falhas na assistência prestada aos hipertensos graves, visando nortear a implementação de um Programa de Hipertensão Arterial do hospital.

**TIPO DE ESTUDO:**

Estudo descritivo transversal.

**LOCAL:**

Ambulatórios de Clínica Médica do Hospital Universitário Clementino Fraga Filho da Universidade Federal do Rio de Janeiro.

**MÉTODOS:**

A população alvo foi composta pelos pacientes hipertensos em acompanhamento no ambulatório de Clínica Médica por um período de quatro meses. Os médicos assistentes obtiveram informações sobre dados demográficos, fatores de risco cardiovascular, lesões de órgãos-alvo, pressão arterial na consulta, terapêutica instituída e adesão do pacie-ente ao tratamento. A distribuição das características dos pacientes foi descrita com as médias e respectivos desvios-padrão e proporções.

**RESULTADOS:**

Dentre os pacientes vistos no período, 24,2% (1.699 pacientes) eram hipertensos, dos quais 65% eram mulheres. A idade média foi de 63,9 anos (desvio-padrão = 11,6). Dislipidemia (49,2%) e diabetes (29,8%) foram os fatores de risco mais encontrados, o órgão alvo mais atingido foi o coração (46,1%) e 70% dos pacientes foram estratificados como de alto risco cardiovascular. Apesar da elevada intensidade de tratamento instituído nos pacientes mais graves (19,4% em um regime de tratamento anti-hipertensivo com três ou mais drogas) o controle da pressão arterial foi baixo (27%). Este estudo orientou a elaboração de uma rotina de diagnóstico e tratamento de hipertensão arterial e norteou os objetivos do Programa de Hipertensão Arterial do Hospital Universitário Clementino Fraga Filho, Universidade Federal do Rio de Janeiro (UCFF-UFRJ), recém-implantado.

**CONCLUSÃO:**

Os pacientes hipertensos em tratamento neste Hospital têm elevado risco cardiovascular e controle inadequado dos níveis tensionais. Faz-se necessário maior esforço para controlar a pressão arterial, pois só assim será possível reduzir a morbimortalidade cardiovascular.

## INTRODUCTION

Cardiovascular diseases remain the leading cause of death in Brazil, reaching 32.3% of all causes of mortality.^1,2,3^ Arterial hyper-tension is the most frequent treatable cardiovascular risk factor for death,^2,3,4^ with prevalence ranging from 10 to 44% in different studies and countries.^4-10^

Without symptoms in its initial stage, hypertension is generally diagnosed only when complications appear, thus causing significant losses in quality of life and increasing mortality rates. The costs for society are high, because this condition requires tertiary care and affects an economically active part of the population.

Seeking to provide the necessary information for the planning of the arterial hypertension program that was to be implemented in the Hospital Universitário Clementino Fraga Filho, Universidade Federal do Rio de Janeiro (HUCFF-UFRJ), hypertensive patients under treatment for at least three months in the internal medicine outpatient unit were evaluated. Data on cardiovascular risk profile (risk factors and target organ damage), blood pressure control, antihypertensive drugs prescribed and compliance with treatment were obtained by means of a standard questionnaire.

## OBJECTIVE

The objective of the study was to characterize the profile of hypertensive patients attended in the internal medicine outpatient unit of the Hospital Universitário Clementino Fraga Filho, Universidade Federal do Rio de Janeiro (HUCFF-UFRJ), in order to implement an arterial hypertension program at the hospital based on:

1. The demand for the treatment of hypertensive outpatients in a University Hospital.
2. The cardiovascular risk profile of these patients.
3. The flaws in the care provided for severely hypertensive patients, in order to improve its quality through the adoption of guidelines.

## MATERIAL AND METHODS

### Type of study

This was a descriptive cross-sectional study based on a census of the hypertensive patients, during the period from September 1998 to January 1999.

### Setting

The study was conducted in the internal medicine outpatient clinic of the Hospital Universitário Clementino Fraga Filho, Universidade Federal do Rio de Janeiro, under the coordination of the Internal Medicine and Epidemiology Units.

### Sample and Procedures

**Figure 1:**
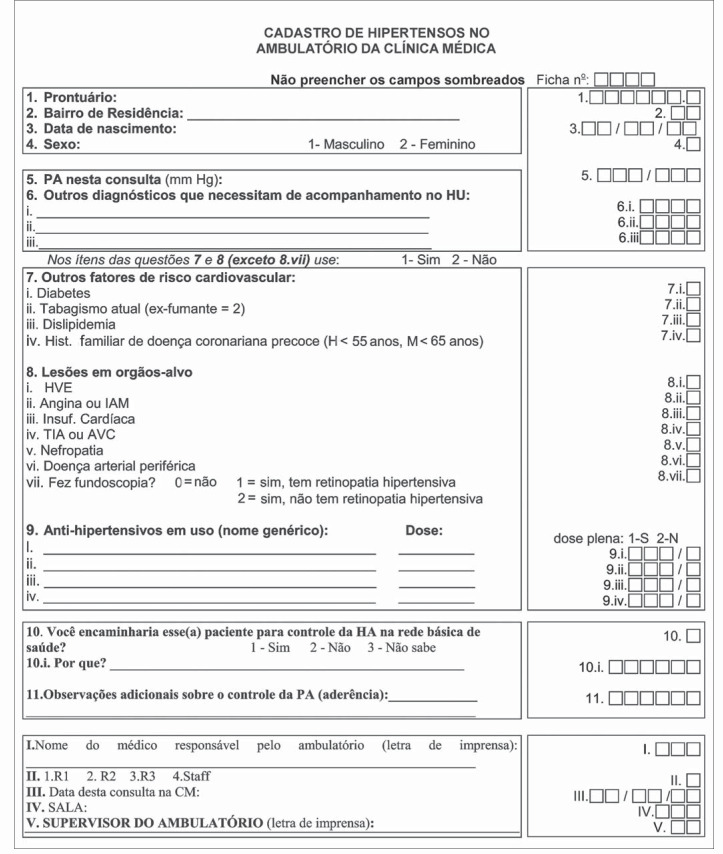
Form for data collection regarding clinical status and cardiovascular risk in patients seen in Hospital Universitário Clementino Fraga Filho, Rio de Janeiro, Brazil.

Data collection forms (Figure 1) were distributed to the 51 residents and 7 senior physicians of the Internal Medicine Unit, who had been previously trained to utilize the questionnaire and measure blood pressure in accordance with the Fourth Brazilian Guidelines on High Blood Pressure.^11^ The mercury column sphygmomanometer was used to measure blood pressure twice during the medical visit and only the second measurement was registered.

All the hypertensive patients^11,12^ who had been under treatment for at least 3 months were eligible to enter the study. Demographic features (gender, date of birth, place of residence), cardiovascular risk factors and the presence of target organ damage^11,12^ were registered. Diabetes, dyslipidemia, family history of early coronary heart disease (men younger than 55 years of age and women younger than 65 years) and current smoking were appraised as risk factors. Target organ damage, such as left ventricular hypertrophy (electrocardiographic and/or echocardiographic criteria), coronary heart disease (angina and/or myocardial infarction), heart failure (clinical and/or echocardiographic criteria), cerebrovascular diseases (stroke and/or history of transient ischemic attacks), hypertensive retinopathy, nephropathy and peripheral arterial disease were evaluated.

Diagnoses of risk factors (diabetes or dyslipidemia) and target organ damage could be based on clinical criteria or confirmed by complementary methods according to the attending physician, following criteria in the literature. The classification of cardiovascular risk was based on the Fourth Brazilian Guidelines on High Blood Pressure,^11^ as follows: risk A – patients without risk factors or target organ damage; risk B – patients with at least one risk factor, except diabetes, and without target organ damage; risk C – patients with diabetes or target organ damage, regardless of the presence of other risk factors.

The rate of hypertension control was defined as the number of treated hypertensive individuals with blood pressure less than 140/90 mmHg, divided by the total number of hypertensive individuals.^9,11,12^

Therapeutic regimens were also registered, as well as any intention to change them, on the part of the physician. The attending physicians also gave their opinions regarding patient compliance with the treatment.

The University Hospital Ethics Committee had previously approved this study protocol.

### Statistical analysis

Means and the respective standard deviations and proportions were used to describe the distribution of the data. The chi-squared test was used to determine the statistical significance of the differences between the proportions and the Student t test was used to compare means.

## RESULTS

A total of 1,699 hypertensive patients were registered, corresponding to 24.2% of all the outpatients attended in the Internal Medicine unit (7,160 patients) during the four-month period.

Women accounted for 65% of the patients, and the average age was 63.9 years (standard deviation = 11.6). Most of the patients (52%) were older than 65 years of age.

Almost half of the patients (47.3%) lived near to the Hospital, while 18.3% came from outside the city of Rio de Janeiro.

### Cardiovascular risk factors

The most frequent risk factor was dyslipidemia (44.2%), followed by diabetes mellitus (29.8%). A family history of coronary heart diseases was present in 18.5% of the patients and only 7.7% were smokers. It was observed that 30.7% of the patients did not present any cardiovascular risk factor, while 37.3% had one risk factor and 27.2% presented two or more. We had no information about risk factors in only 4.8% of the patients.

### Target organ damage

In 39.2% of the patients, no target organ involvement was diagnosed, while 29.4% had one damaged organ and 25.2% had two or more. Heart involvement was the most frequent finding (46.1%), followed by cerebrovascular disorders (30.8%), hypertensive nephropathy (11.1%) and peripheral arterial disease (8.4%).

Left ventricular hypertrophy was the most common heart damage (33.9%), followed by coronary heart disease (20.1%) and heart failure (10.2%). Hypertensive retinopathy was registered in 22.3% of patients. However, in 52.1% of the patients, funduscopic examination was not performed at all.

In the cardiovascular risk classification, 70% of the patients were included in cardiovascular risk category C, 14.9% in category B and 15.1% in category A.

### Antihypertensive therapeutics

**Figure 2:**
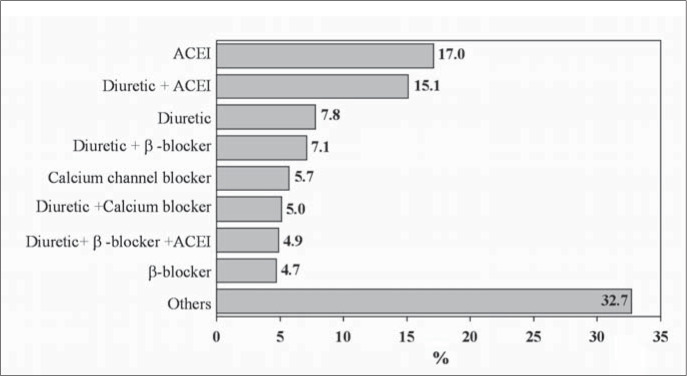
Most commonly used anti-hypertensive therapeutic regimens among patients seen in Hospital Universitário Clementino Fraga Filho, Rio de Janeiro, Brazil. ACEI = angiotensin-conversion enzyme inhibitors, β-blockers = beta-blockers.

Diuretics were the pharmacological group that was prescribed most (33.5%): mainly hydrochlorothiazide. Angiotensin-converting enzyme inhibitors were used in 31.2% of the patients, and captopril was the most frequently prescribed of these. Calcium channel blockers and beta-blockers were prescribed with the same frequency (14.5%), and nifedipine and propranolol were the drugs most used in each group. Figure 2 presents the most common therapeutic regimens.

Monotherapy was prescribed for 36.3% of the patients, while 41.9% used two drugs and 19.4% used three or more antihypertensive drugs. Only 2.4% of the patients were solely on non-pharmacological treatment.

**Figure 3:**
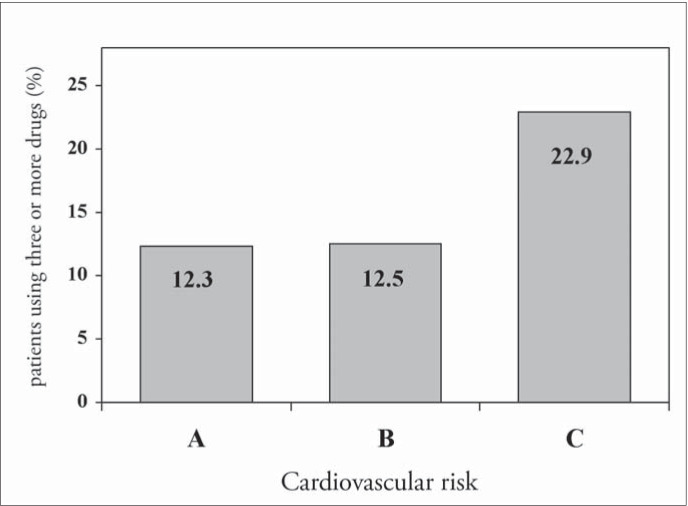
Treatment intensity at the different levels of cardiovascular risk in patients seen in Hospital Universitário Clementino Fraga Filho, Rio de Janeiro, Brazil.

**Figure 4:**
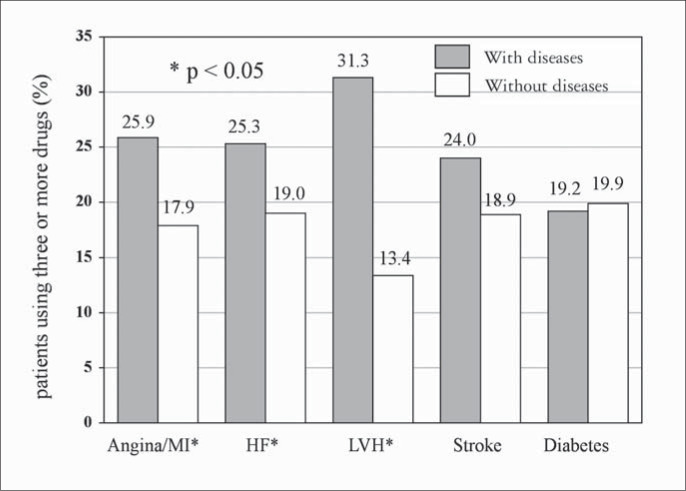
Treatment intensity according to the presence of different target organs damaged and diabetes in patients seen in Hospital Universitário Clementino Fraga Filho, Rio de Janeiro, Brazil. MI = myocardial infarction. HF = heart failure. LVH = left ventricular hypertrophy.

High cardiovascular risk and the presence of target organ damage, especially heart involvement, determined the intensity of treatment (number of antihypertensive drugs prescribed). However, the same was not observed with the diabetic hypertensive patients (Figures 3 and 4).

The attending physicians considered that 55.1% of the patients were complying with regular use of the antihypertensive medications.

### Blood pressure control

**Figure 5:**
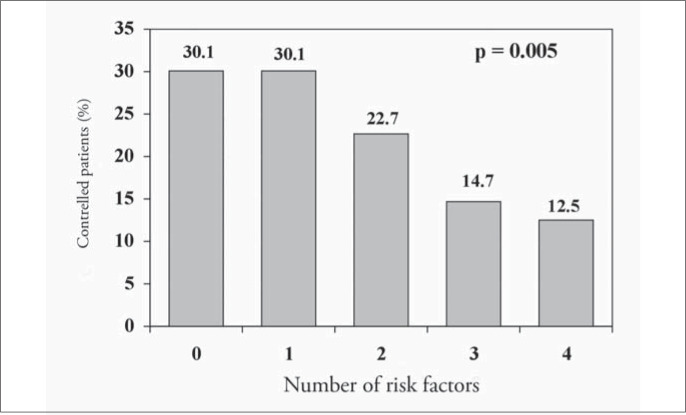
Blood pressure control rate according to the number of cardiovascular risk factors in patients seen in Hospital Universitário Clementino Fraga Filho, Rio de Janeiro, Brazil..

**Figure 6:**
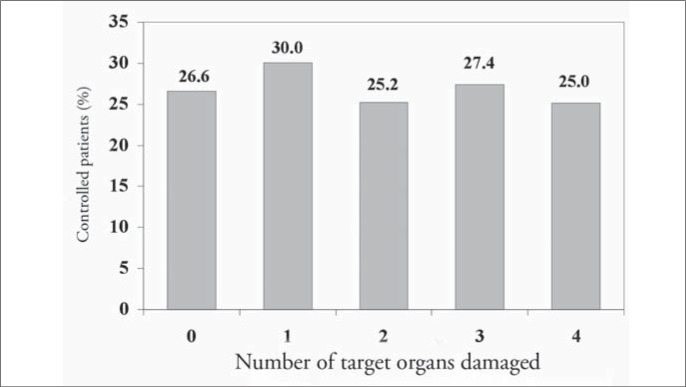
Blood pressure control rate according to the number of target organs damaged in patients seen in Hospital Universitário Clementino Fraga Filho, Rio de Janeiro, Brazil.

**Figure 7:**
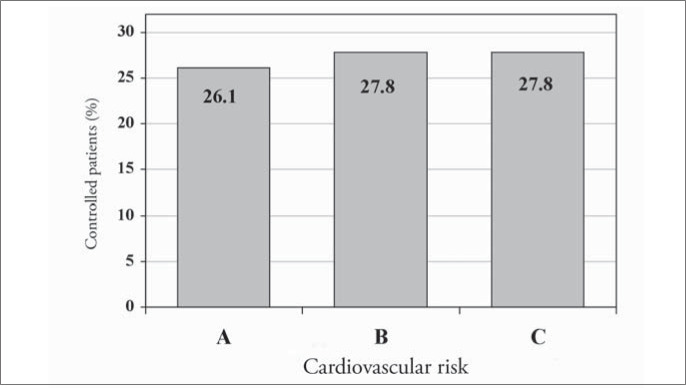
Blood pressure control rate according to the cardiovascular risk stratification in patients seen in Hospital Universitário Clementino Fraga Filho, Rio de Janeiro, Brazil.

**Figure 8:**
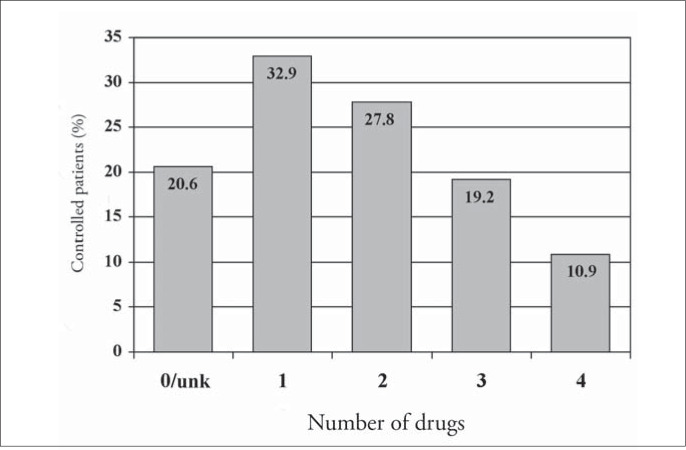
Blood pressure control rate according to the treatment intensity in patients seen in Hospital Universitário Clementino Fraga Filho, Rio de Janeiro, Brazil. Unk = unknown.

Of the 1,699 hypertensive patients, 27% had their condition under control. The control rate was similar in both genders (p = 0.29). However, the controlled patients were older than uncontrolled ones (68.2 compared with 62.3 years; p < 0.001). The attending physicians reported that they intended to change the antihypertensive medications in only 20.1% (242) of the patients with blood pressure levels higher than 140 x 90 mmHg. Figures 5 to 8 present the blood pressure control rates by number of risk factors, target organ damage, cardiovascular risk stratification and treatment intensity. Resistant hypertension (patients with uncontrolled blood pressure in spite of the use of three or more antihypertensive drugs with different mechanisms of action)^13^ was diagnosed in 257 patients (15.1%).

The majority of the blood pressure records (93.4%) made approximations of the values to multiples of 5 or 10.

## DISCUSSION

The evolution of epidemiological, clinical and technological knowledge has given the possibility of interventions at different levels of prevention and treatment, that are capable of reducing cardiovascular morbidity and mortality.^13-16^

This study was a tool for estimating the prevalence and severity of hypertension in the outpatient clinic unit of Hospital Universitário Clementino Fraga Filho, Universidade Federal do Rio de Janeiro, thereby allowing evaluation to be made of the quality and effectiveness of the health care provided. These results contributed towards planning the implementation of a local hypertension program.

The observed prevalence of arterial hyper-tension was similar to what has been found in population-based studies in Brazil^5-8^ and other countries.^4,9,10^ High prevalence of dyslipidemia and diabetes^14-16^ was found, and heart involvement was the most common end-organ damage, especially left ventricular hypertrophy (33.9%)

Of the registered patients, 70% were classified as being at high cardiovascular risk, a patient profile that requires tertiary care. These findings guided the objectives of the hyper-tension program.

The way in which the blood pressure was recorded in the present study reflects a reality of the health services. Despite the training given in blood pressure measurement, a strong tendency to approximate the values to multiples of 10 or 5 was observed, which compromised the correct evaluation of blood pressure control.

The therapeutic regimens most frequently prescribed (Figure 2) were appropriate, according to recent guidelines,^11,12^ and the highest intensity of treatment was found in patients with heart involvement and cardiovascular risk C (Figures 3 and 4). Nevertheless, only 27% of the hypertensive patients achieved blood pressure control, much lower than the desirable rate. This is similar to findings from other studies done in Brazil^6,8^ and developed countries,^9,10^ and it demonstrates the difficulty in achieving such a goal, which requires a search for new strategies and a change of approach to arterial hypertension.

The higher the number of risk factors was, the poorer the hypertension control (Figure 5), probably because of the metabolic conditions associated. Otherwise, blood pressure control was independent of the number of target organs damaged (Figure 6) and it was similar at different levels of cardiovascular risk: risk A (26.1%), risk B (27.8%) and risk C (27.8%) (Figure 7), although the intensity of treatment was higher among high-risk patients (Figure 3).

The attending physicians reported an intention to change therapeutic regimens in relation to only 20% of the uncontrolled patients. The importance of a more rigorous control of blood pressure may be neglected when blood pressure levels are partially reduced. The number of drugs was inversely related to blood pressure control, maybe because greater numbers of drugs decreased the compliance with therapy (Figure 8). An even larger effort is necessary, to make sure that physicians understand the need for better control over blood pressure, since this is the only way to achieve a significant reduction in the morbidity and mortality rates for cardiovascular diseases.

Optimized blood pressure control is essential for cardiovascular protection. The short-term priorities for achieving such a goal should be: enhancement of the accessibility of medical care, more rigorous adherence to the guidelines for diagnosis and treatment of high blood pressure, and stricter compliance with therapy.^10^

On the basis of such aims and the results from the present study, the hypertension program at the Hospital Universitário Clementino Fraga Filho was implemented in 1999, with the following purposes:

1. To standardize the routine for the diagnosis and treatment of hypertension in the hospital, thereby allowing for continuous evaluation and the construction of indicators to reflect the effectiveness and quality of the attendance;
2. To improve cardiovascular risk stratification through the establishment of a specific investigation routine, including funduscopy, ambulatory blood pressure monitoring and 24-hour microalbuminuria evaluation.
3. To improve blood pressure control through a multidisciplinary approach towards patient follow-up, in order to increase compliance with antihypertensive treatment.
4. To offer training for undergraduate and postgraduate students in clinical and epidemiological areas related to hypertension. 5. To create a special outpatient service for resistant hypertension, so as to improve follow-up and research new approaches towards decreasing cardiovascular morbidity and mortality. These include the construction of an information system that allows the investigation of associations between clinical and demographic variables and cardiovascular outcomes in this special group of resistant hypertensive patients.

The problems observed in the investigation and treatment of hypertensive patients that led to these findings has resulted in the drafting of guidelines for the diagnosis, treatment and management of arterial hypertension, which are now in their fourth edition. Also, a new care unit for patients with resistant hypertension was created to study better approaches for their follow-up and treatment. Research in this area is increasing and contributing towards better understanding of this complex health problem.

## CONCLUSIONS

This large descriptive cross-sectional study among outpatients attending a university tertiary-care hospital showed that hypertensive patients had a profile of high cardiovascular risk and low blood pressure control rate.

The hypertension program of Hospital Universitário Clementino Fraga Filho is making great efforts towards improving the compliance of physicians and other health personnel with the hypertension guidelines, in order to improve blood pressure control. This is the only way to achieve a significant reduction in the morbidity and mortality rates for cardiovascular diseases.
